# A Network-Guided Approach to Discover Phytochemical-Based Anticancer Therapy: Targeting MARK4 for Hepatocellular Carcinoma

**DOI:** 10.3389/fonc.2022.914032

**Published:** 2022-07-22

**Authors:** Sarfraz Ahmed, Mohammad Mobashir, Lamya Ahmed Al-Keridis, Nawaf Alshammari, Mohd Adnan, Mohammad Abid, Md Imtaiyaz Hassan

**Affiliations:** ^1^ Department of Biosciences, Faculty of Natural Science, Jamia Millia Islamia, New Delhi, India; ^2^ Centre for Interdisciplinary Research in Basic Sciences, Jamia Millia Islamia, New Delhi, India; ^3^ Department of Biology, College of Science, Princess Nourah Bint Abdulrahman University, Riyadh, Saudi Arabia; ^4^ Department of Biology, College of Science, University of Hail, Hail, Saudi Arabia

**Keywords:** MARK4, HCC, potential genes, herbal drugs, signaling pathways, clinical relevance, biological networks

## Abstract

MAP/microtubule affinity-regulating kinase 4 (MARK4) is associated with various biological functions, including neuronal migration, cell polarity, microtubule dynamics, apoptosis, and cell cycle regulation, specifically in the G1/S checkpoint, cell signaling, and differentiation. It plays a critical role in different types of cancers. Hepatocellular carcinoma (HCC) is the one of the most common forms of liver cancer caused due to mutations, epigenetic aberrations, and altered gene expression patterns. Here, we have applied an integrated network biology approach to see the potential links of MARK4 in HCC, and subsequently identified potential herbal drugs. This work focuses on the naturally-derived compounds from medicinal plants and their properties, making them targets for potential anti-hepatocellular treatments. We further analyzed the HCC mutated genes from the TCGA database by using cBioPortal and mapped out the MARK4 targets among the mutated list. MARK4 and Mimosin, Quercetin, and Resveratrol could potentially interact with critical cancer-associated proteins. A set of the hepatocellular carcinoma altered genes is directly the part of infection, inflammation, immune systems, and cancer pathways. Finally, we conclude that among all these drugs, Gingerol and Fisetin appear to be the highly promising drugs against MARK4-based targets, followed by Quercetin, Resveratrol, and Apigenin.

## Introduction

Cancer, often known as a malignant tumor, is a category of diseases characterized by abnormal cell proliferation with the ability to invade and spread to other parts of the body. Cancer has six basic capabilities to enable the development of human tumors: sustaining proliferative signals, avoiding growth suppressors, resisting cell death, enabling replicative immortality, initiating angiogenesis, and activating invasion and metastasis are six basic capabilities that cancer has to enable the development of human tumors (1–4). Cancer is one of the leading causes of death worldwide, with increasing number of cancer patients. Cancers are classified into several categories depending on their origin and form. Hepatocellular carcinoma (HCC) is one of them, and it is the fifth most common cancer in men and the seventh most common cancer in women. The majority of the disease burden is carried by developing countries, with the greatest incidence rates observed in areas where hepatitis B virus (HBV) infection is endemic. Infection with HBV or HCV, alcoholic liver disease, and most likely nonalcoholic fatty liver disease are major risk factors for HCC. Hereditary hemochromatosis, alpha1-antitrypsin deficiency, autoimmune hepatitis, certain porphyrias, and Wilson’s disease are less prevalent causes (5–9). Gene expression changes, mutations in coding and non-coding regions, and epigenetic changes are among the most common changes (1, 10–14).

Despite significant efforts, cancer continues to be a great challenge for global health experts. Surgery, chemotherapy, and radiotherapy are the most common and effective cancer treatments (15–17). These treatments, however, have many limitations. Because of poor diagnosis and other circumstances, most cancer patients are detected too late to undergo surgery. As a result, there is a continuing desire for innovative, effective, and affordable anti-cancer medications (18–20). In many countries throughout the world, medicinal plants are used as a cancer therapy option (21–23). Medicinal plants have been utilized in medicine from the dawn of civilization, as evidenced by ancient scripts and traditional herbal medicine recipes (24–27). Despite the long history of plants used as medicinal, pharmaceutical companies are less interested in phytochemical research than in synthetic drug development. The lack of information regarding plant-based medical treatments is primarily responsible for researchers’ interest in natural goods as prospective drugs for harmful diseases such as cancer.

Microtubule-Affinity Regulated Kinase 4 (MARK4) is a Ser/Thr kinase part of the AMPK family that regulates microtubule dynamics by phosphorylating microtubule-related proteins. MARK4 is encoded by genes located on chromosome 19 of the human genome (28–32). MARK4 controls cell division, proliferation, and cell cycle control *via* regulating microtubule dynamics through phosphorylation. MARK4 is divided into two isoforms: MARK4S and MARK4L, which result from alternative splicing (32–34). MARK4S is a short protein with 18 exons that codes for a mature polypeptide with 688 amino acid residues. MARK4L, on the other hand, loses exon 16, which causes a downstream stop codon to shift, resulting in the production of a longer polypeptide with 752 amino acid residues. Both proteins have the same kinase domain, but their C-terminal tails are different. MARK4 is overexpressed in various metabolic conditions, including diet-induced obesity, cardiovascular disease, type II diabetes, Alzheimer’s disease, hepatocellular carcinoma, glioma, and metastatic breast carcinomas, etc. It’s overexpressed in cancerous cells, including hepatocellular carcinoma and leukemia, and associated with breast and prostate cancer growth.

Furthermore, overexpression of MARK4 causes hyperphosphorylation of tau protein, which leads to the tauopathies (32, 35, 36). According to previous detailed investigations, MARK4 is thought to play significant roles in guiding neuronal migration, cell polarity, microtubule dynamics, apoptosis, cell cycle regulation, specifically in the G1/S checkpoint, cell signaling, and differentiation (32, 33, 35).

MARK4 plays a crucial role in energy metabolism and homeostasis. Any changes in MARK4 expression can disrupt important cellular pathways such as mTOR and NF-kB, resulting in a wide range of health issues. MARK4 is an important component of the Wnt signaling system, and linked to Wnt-induced prostate cancer. MARK4 promotes adiposity and cell death by activating JNK1 and inhibiting the p38MAPK pathways. Blocking Hippo signaling also stimulates breast cancer cell proliferation and migration (29, 35, 37, 38).

Overexpression of MARK4 is linked to malignancies like metastatic transitions, aberrant and uncontrolled neuronal migrations, and disruption of microtubule dynamics. MARK4 is linked to breast cancer growth and metastasis *via* Hippo signaling. Inhibiting MARK4 expression slows the progression of gliomas. MARK4 inhibitors limit the growth and proliferation of various cancer cell types, suggesting that they may be effective in improving cancer outcomes. Glioblastomas and prostate cancer have high levels of this kinase, thus considered as an attractive therapeutic target for cancer, diabetes, and neurological disorders (25, 30, 39, 45). These inhibitors reduce the growth and proliferation of various cancer cell types, highlighting the importance of MARK4 inhibitors in improving the outcomes of malignancies. MARK4 has demonstrated to increase microtubule dynamics and confer paclitaxel resistance in HCC, making it a suitable target for treating paclitaxel resistance. MARK4 is a viable target for sensitizing HCC to paclitaxel treatment because it can create a direct bond with microtubules.

We focused on gene expression and mutational profiling of HCC, establishing a relationship between MARK4-associated proteins, docking analysis of natural products against probable protein targets, and network-level comprehension of the essential genes/proteins mentioned in this study. Our focus to establish the role of MARK4 in HCC. In addition to see therapeutic potential of vanillin in targeting MARK4 associated diseases. Furthermore, we investigated the overall influence of vanillin and MARK4 on the important genes and major signaling pathways linked to HCC.

## Materials and Methods

### Data Collections and Preparations

**Figure 1:**
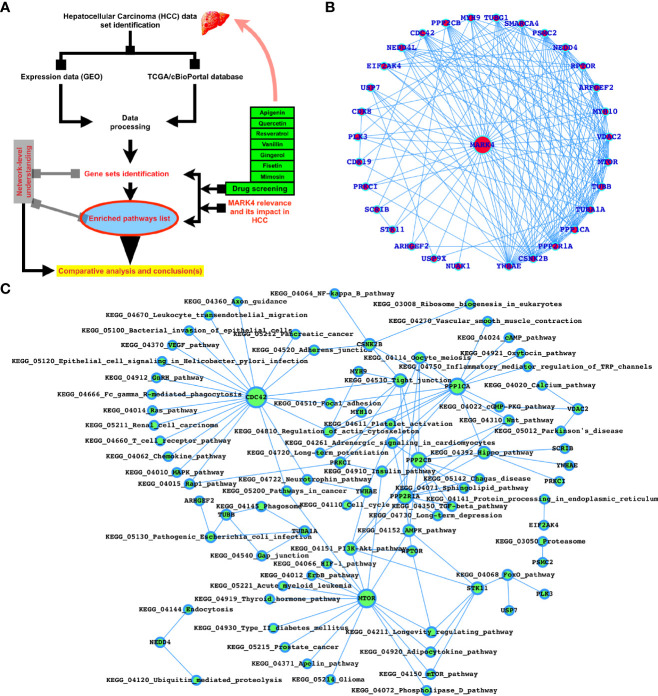
MARK4-interactors profiling and potential drugs target properties. **(A)** Workflow of this study. **(B)** Top-ranked MARK4 interactors. **(C)** MARK4-associated pathways.

All of the steps in this investigation are summarized in **Figure 1A**. Using the FunCoup network database, we gathered all of the proteins interacting with MARK4 and screened the top interactors. The linked KEGG pathways with top-interactors were predicted, and the overall enriched KEGG pathways for all MARK4 interactors. Using SwissTargetPrediction, we mapped out all the proteins that may bind with the natural products employed in this investigation and determined their physicochemical properties (46–49) using SwissADME. SwissTargetPrediction is a web tool, that aimed to predict the most probable protein targets of small molecules. Reverse screening is used to make predictions based on the similarity principle. The 2019 version is described here, and it represents a significant improvement in terms of underlying data, back-end, and web interface. The model was retrained, and the similarity thresholds were revised once the bioactivity data was refreshed. The predictions in the updated edition are made by looking for similar molecules in 2D and 3D within a larger collection of chemicals that have been experimentally active on a wider selection of large number of macromolecular targets. An efficient back-end implementation can speed up returning findings for a drug-like compound on human proteins. Absorption, distribution, metabolism, and excretion (ADME) testing is essential for drug development process when the number of potential compounds is expanding. The new SwissADME web application provides access to a pool of rapid yet reliable predictive models for physicochemical properties, pharmacokinetics, drug-likeness, and medicinal chemistry friendliness.

### Docking Analysis

After calculating the individual-level properties, we have performed docking for the combined protein-target against the selected herbal drugs, using SwissDock (18, 48, 50, 51). The herbal compound structures were obtained from the PubChem database (52–55).

### Clinical Relevance and Network-Level Analysis

We have used cBioPortal to access TCGA datasets of human HCC samples to analyze the mutated genes, and prepare the list of mutated genes common to MARK4 interactors and predicted the enriched KEGG pathways (56–59). After predicting mutated genes and enriched pathways, the next step was to understand the network and the connections between the genes inside the DEG network. The network was shown using Cytoscape (60) and the FunCoup database (51, 61–66). MATLAB coding was used for figure plotting and analysis. The primary idea of the FunCoup network database to predict four different forms of functional coupling or links, such as protein complexes, protein-protein physical interactions, metabolic, and signaling pathways (51, 61, 64, 65, 67–70). Swiss-dock was utilized for docking analysis (http://www.swissdock.ch) (18, 48), with PubChem (https://pubchem.ncbi.nlm.nih.gov), UniProt (https://www.uniprot.org/), and swiss-model (https://swissmodel.expasy.org) as supporting databases (18). We calculated binding free energy from docking analysis. The network-level and required analytic scripts and the findings, which comprised the number of connections per gene and the genes belonging to various pathways, were developed in MATLAB (50).

## Results

### Potential MARK4 Proteins and the Physicochemical Properties of Herbal Drugs

Using the protein-protein interaction (PPIs) database, we predicted MARK4 related proteins, physicochemical properties using swissADME, and herbal drug possible target proteins using SwissTargetPrediction in the first phase. In **Figure 1A**, the extremely top-ranked (based on PPI scores > 0.8 to 1.0 (highest score)) PPIs of MARK4 were presented and we observe that YWHAE, CSNK2B, PPP2R1A, PPP1CA, TUBA1A, TUBB, MTOR, VDAC2, MYH10, ARFGEF2, RPTOR, NEDD4, PSMC2, SMARCA4, TUBG1, MYH9, PPP2CB, CDC42, NEDD4L, EIF2AK4, USP7, CDK8, PLK3, PRKCI, SCRIB, STK11, ARHGEF2, USP9X, and NUAK1 were among them and YWHAE, CSNK2B, PPP2R1A, PPP1CA, TUBA1A, TUBB, MTOR, VDAC2, MYH10, ARFGEF2, RPTOR, and NEDD4 are those proteins which connect the highest number of proteins listed here (**Figure 1B**). As shown in **Figure 1C**, most of these proteins are associated with critical signaling pathways and biological functions. The majority of these pathways are known to control various types of cancers, including HCC directly. A detailed **Table 1** was presented to display the MARK4-interactors-associated pathways.

**Table 1:** MARK4-interactors-associated pathways.

NEDD4	KEGG_04120_Ubiquitin_mediated_proteolysis
NEDD4	KEGG_04144_Endocytosis
CDC42	KEGG_04010_MAPK_pathway
CDC42	KEGG_04062_Chemokine_pathway
CDC42	KEGG_04360_Axon_guidance
CDC42	KEGG_04370_VEGF_pathway
CDC42	KEGG_04510_Focal_adhesion
CDC42	KEGG_04520_Adherens_junction
CDC42	KEGG_04530_Tight_junction
CDC42	KEGG_04660_T_cell_receptor_pathway
CDC42	KEGG_04666_Fc_gamma_R-mediated_phagocytosis
CDC42	KEGG_04670_Leukocyte_transendothelial_migration
CDC42	KEGG_04722_Neurotrophin_pathway
CDC42	KEGG_04810_Regulation_of_actin_cytoskeleton
CDC42	KEGG_04912_GnRH_pathway
CDC42	KEGG_05100_Bacterial_invasion_of_epithelial_cells
CDC42	KEGG_05120_Epithelial_cell_signaling_in_Helicobacter_pylori_infection
CDC42	KEGG_05200_Pathways_in_cancer
CDC42	KEGG_05211_Renal_cell_carcinoma
CDC42	KEGG_05212_Pancreatic_cancer
MYH9	KEGG_04530_Tight_junction
MYH9	KEGG_04810_Regulation_of_actin_cytoskeleton
PPP2CB	KEGG_04114_Oocyte_meiosis
PPP2CB	KEGG_04310_Wnt_pathway
PPP2CB	KEGG_04350_TGF-beta_pathway
PPP2CB	KEGG_04530_Tight_junction
PPP2CB	KEGG_04730_Long-term_depression
PPP2CB	KEGG_05142_Chagas_disease
PPP2R1A	KEGG_04114_Oocyte_meiosis
PPP2R1A	KEGG_04310_Wnt_pathway
PPP2R1A	KEGG_04350_TGF-beta_pathway
PPP2R1A	KEGG_04530_Tight_junction
PPP2R1A	KEGG_04730_Long-term_depression
PPP2R1A	KEGG_05142_Chagas_disease
YWHAE	KEGG_04110_Cell_cycle
YWHAE	KEGG_04114_Oocyte_meiosis
YWHAE	KEGG_04722_Neurotrophin_pathway
ARHGEF2	KEGG_05130_Pathogenic_Escherichia_coli_infection
STK11	KEGG_04150_mTOR_pathway
STK11	KEGG_04920_Adipocytokine_pathway
MYH10	KEGG_04530_Tight_junction
MYH10	KEGG_04810_Regulation_of_actin_cytoskeleton
PSMC2	KEGG_03050_Proteasome
PRKCI	KEGG_04530_Tight_junction
PRKCI	KEGG_04910_Insulin_pathway
VDAC2	KEGG_04020_Calcium_pathway
VDAC2	KEGG_05012_Parkinson’s_disease
TUBA1A	KEGG_04540_Gap_junction
TUBA1A	KEGG_05130_Pathogenic_Escherichia_coli_infection
PPP1CA	KEGG_04114_Oocyte_meiosis
PPP1CA	KEGG_04270_Vascular_smooth_muscle_contraction
PPP1CA	KEGG_04510_Focal_adhesion
PPP1CA	KEGG_04720_Long-term_potentiation
PPP1CA	KEGG_04810_Regulation_of_actin_cytoskeleton
PPP1CA	KEGG_04910_Insulin_pathway
TUBB	KEGG_04540_Gap_junction
MTOR	KEGG_04012_ErbB_pathway
MTOR	KEGG_04150_mTOR_pathway
MTOR	KEGG_04910_Insulin_pathway
MTOR	KEGG_04920_Adipocytokine_pathway
MTOR	KEGG_04930_Type_II_diabetes_mellitus
MTOR	KEGG_05200_Pathways_in_cancer
MTOR	KEGG_05214_Glioma
MTOR	KEGG_05215_Prostate_cancer
MTOR	KEGG_05221_Acute_myeloid_leukemia
CSNK2B	KEGG_04310_Wnt_pathway
CSNK2B	KEGG_04520_Adherens_junction
CSNK2B	KEGG_04530_Tight_junction
TUBB	KEGG_04540_Gap_junction
TUBB	KEGG_05130_Pathogenic_Escherichia_coli_infection
PRKCI	KEGG_04392_Hippo_pathway
PPP2CB	KEGG_04392_Hippo_pathway
PPP2R1A	KEGG_04392_Hippo_pathway
PPP1CA	KEGG_04392_Hippo_pathway
SCRIB	KEGG_04392_Hippo_pathway
YWHAE	KEGG_04392_Hippo_pathway
CSNK2B	KEGG_03008_Ribosome_biogenesis_in_eukaryotes
EIF2AK4	KEGG_04141_Protein_processing_in_endoplasmic_reticulum
CDC42	KEGG_04014_Ras_pathway
PRKCI	KEGG_04015_Rap1_pathway
CDC42	KEGG_04015_Rap1_pathway
MTOR	KEGG_04371_Apelin_pathway
CSNK2B	KEGG_04064_NF-kappa_B_pathway
CSNK2B	KEGG_04064_NF-kappa_B_pathway
CSNK2B	KEGG_04064_NF-kappa_B_pathway
CSNK2B	KEGG_04064_NF-kappa_B_pathway
CSNK2B	KEGG_04064_NF-kappa_B_pathway
CSNK2B	KEGG_04064_NF-kappa_B_pathway
CSNK2B	KEGG_04064_NF-kappa_B_pathway
MTOR	KEGG_04066_HIF-1_pathway
PLK3	KEGG_04068_FoxO_pathway
STK11	KEGG_04068_FoxO_pathway
USP7	KEGG_04068_FoxO_pathway
MTOR	KEGG_04072_Phospholipase_D_pathway
PPP2CB	KEGG_04071_Sphingolipid_pathway
PPP2R1A	KEGG_04071_Sphingolipid_pathway
PPP1CA	KEGG_04024_cAMP_pathway
PPP1CA	KEGG_04022_cGMP-PKG_pathway
VDAC2	KEGG_04022_cGMP-PKG_pathway
MTOR	KEGG_04151_PI3K-Akt_pathway
PPP2CB	KEGG_04151_PI3K-Akt_pathway
PPP2R1A	KEGG_04151_PI3K-Akt_pathway
RPTOR	KEGG_04151_PI3K-Akt_pathway
STK11	KEGG_04151_PI3K-Akt_pathway
YWHAE	KEGG_04151_PI3K-Akt_pathway
YWHAE	KEGG_04151_PI3K-Akt_pathway
MTOR	KEGG_04152_AMPK_pathway
PPP2CB	KEGG_04152_AMPK_pathway
PPP2R1A	KEGG_04152_AMPK_pathway
RPTOR	KEGG_04152_AMPK_pathway
STK11	KEGG_04152_AMPK_pathway
TUBB	KEGG_04145_Phagosome
TUBA1A	KEGG_04145_Phagosome
PPP1CA	KEGG_04750_Inflammatory_mediator_regulation_of_TRP_channels
MTOR	KEGG_04211_Longevity_regulating_pathway
RPTOR	KEGG_04211_Longevity_regulating_pathway
STK11	KEGG_04211_Longevity_regulating_pathway
PPP1CA	KEGG_04611_Platelet_activation
PRKCI	KEGG_04611_Platelet_activation
PPP1CA	KEGG_04921_Oxytocin_pathway
MTOR	KEGG_04919_Thyroid_hormone_pathway
PPP1CA	KEGG_04261_Adrenergic_signaling_in_cardiomyocytes
PPP2CB	KEGG_04261_Adrenergic_signaling_in_cardiomyocytes
PPP2R1A	KEGG_04261_Adrenergic_signaling_in_cardiomyocytes

**Figure 2:**
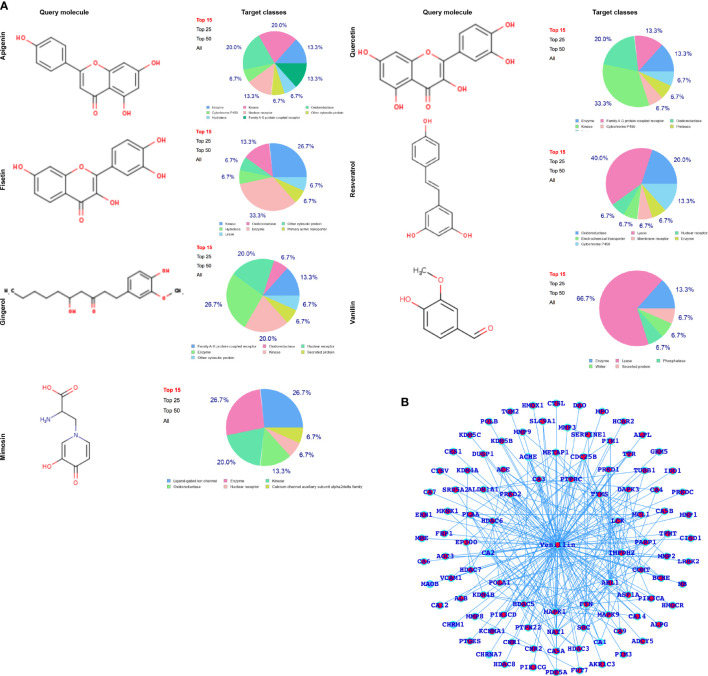
**(A)** Selected herbal drugs and their potential proteins interact with the respective drugs. Here, the pie chart represents the classes of the proteins. **(B)** Vanillin-associated proteins.

**Figure 3:**
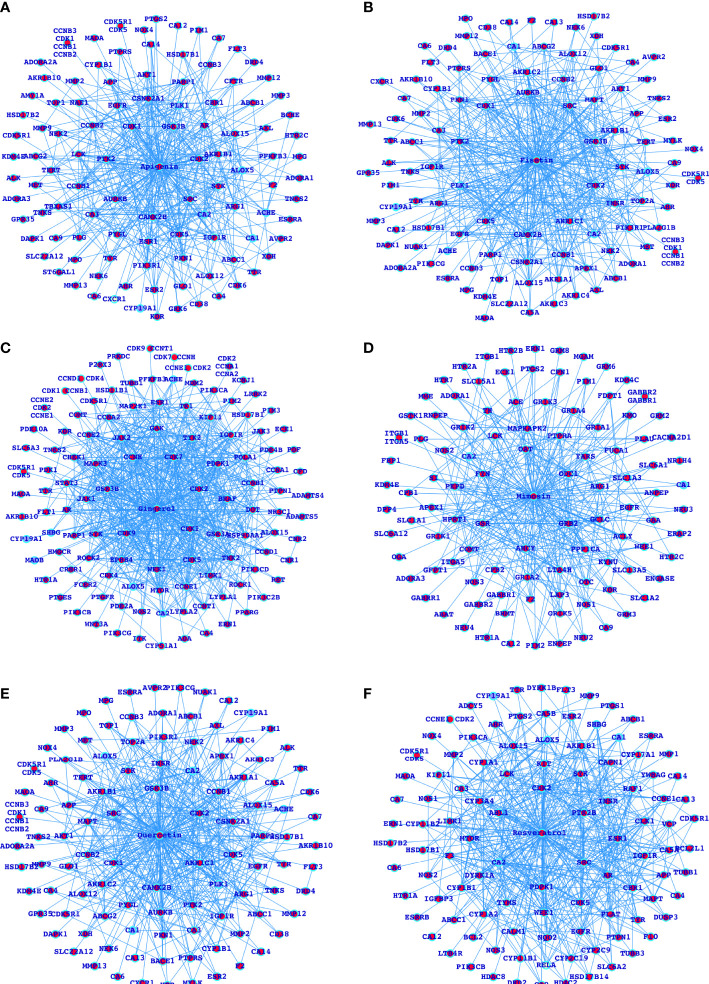
Networks for the selected herbal drugs and their potential proteins interacting with the respective drugs **(A-F)**.

After performing SwissADME (Physico-chemical properties study) analysis, as shown in **Figure 2A**, we have predicted the potential protein targets by using SwissTarget Prediction (**Figures 2B** and **3A–F**) and performed the docking of the MARK4 interactors proteins and the respective herbal drugs interactors (**Figure 3**) and have presented the delta G (ΔG) of all the combinations (drug versus MARK4 interactor or protein) in **Table 2**.

**Table 2:** Docking of the MARK4 interactors proteins and the respective interactors (small molecule inhibitors).

Apigenin		Fisetin		Gingerol	
Proteins	Δ*G*	Proteins	Δ*G*	Proteins	Δ*G*
ABCC1	ND	ABCC1	ND	BRAF	ND
AURKB	-7.62	AURKB	-8.16	CCNH	-7.36
CAMK2B	ND	CAMK2B	ND	CCNT1	-7.8
CDK1	-7.49	CDK1	-7.54	CDK1	-8.17
CDK2	-7.8	CDK2	-7.97	CDK2	-8.34
CDK5	-8.07	CDK5	-8.45	CDK5	-8.1
CSNK2A1	-7.83	CSNK2A1	ND	CDK7	-7.99
GSK3B	-7.51	GSK3B	-7.51	CDK9	-7.94
PKN1	-7.29	MAPT	-6.52	EPHB4	-7.5
PTK2	-7.54	PKN1	-7.36	GAK	-7.51
PTPRS	-7.92	PTK2	-8.08	GSK3A	-7.88
SRC	-7.87	PTPRS	-8.33	GSK3B	-7.9
SYK	-7.4	SRC	-7.77	JAK1	-8.61
		SYK	-7.4	JAK2	-8.51
				LIMK1	-8.13
				MAPK3	-9.1
				PDPK1	-8.12
				SYK	-7.73
				TNK2	-7.98
				TYK2	-8.33
				WEE1	-7.99
Quercetin		Resveratrol		Vanillin	
Proteins	Δ*G*	Proteins	Δ*G*	Proteins	Δ*G*
ABCC1	ND	ABCC1	ND	ABL1	-6.59
AURKB	-7.94	ABL1	-7.91	FYN	-6.35
CAMK2B	ND	CDK2	-7.44	HDAC5	ND
CDK1	-7.5	CDK5	-7.49	KDM4B	-6.58
CDK2	-7.99	CLK1	-7.65	MAPK1	-6.47
CDK5	-8.46	DYRK1A	-7.28	MAPK9	-6.38
CSNK2A1	-8.27	DYRK1B	-7.63	MB	-6.14
GSK3B	-8.03	KIT	-7.26	PRKD2	ND
MAPT	-6.41	LIMK1	-7.66	SRC	-6.27
PKN1	-7.41	MAPT	-6.37		
PTK2	-7.43	NQO2	ND		
PTPRS	-8.27	PDPK1	-7.61		
SRC	-7.66	SRC	-7.47		
SYK	-7.66	SYK	-7.55		
		WEE1	-7.48		

ND, Not determined

### Functional Profiling and Alteration as a Result of Developing Normally to HCC

**Figure 4:**
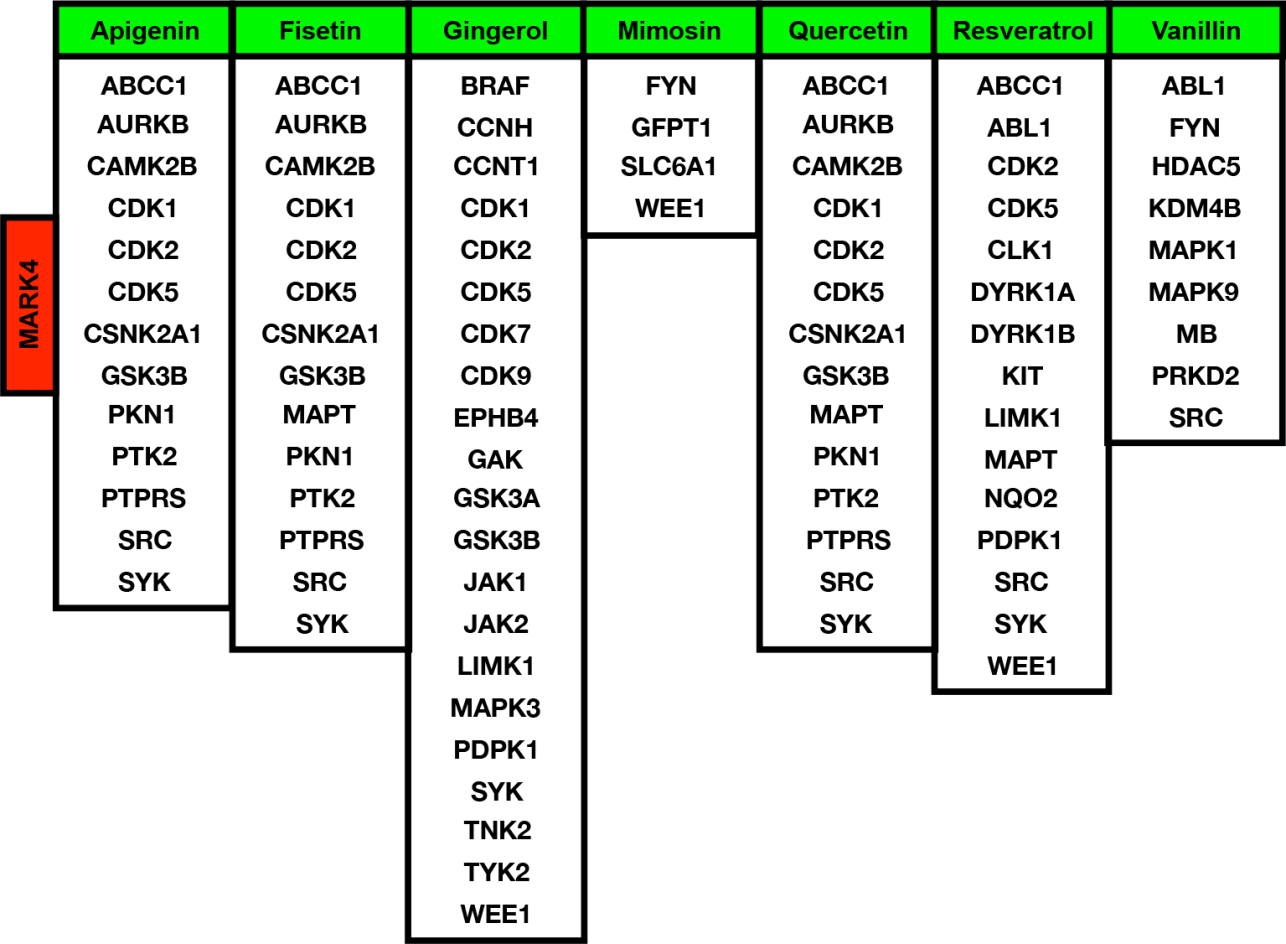
Proteins are common with the selected herbal drug target proteins interacting.

After analyzing the MARK4-associated proteins and the biological pathways, we have performed the swissADME of the selected potential herbal drugs (**Figure 2D**) and have predicted potential protein targets by using SwissTargetPrediction (**Figures 2B** and **3A–F**). These potential herbal drugs were Apigenin, Fisetin, Gingerol, Mimosin, Quercetin, Resveratrol, and Vanillin. Further, we have also mapped out the proteins that were the target proteins for the respective drugs and were common with MARK4 protein targets (**Figure 4**). Here, MARK4 shares the highest number of the target protein with Gingerol, followed by Resveratrol, Quercetin, Fisetin, Apigenin, and Vanillin. In contrast, Mimosin shares the list number of the target proteins. Now, we have performed all the docking profiling with drug—target combinations except the drug Mimosin and the reasons for not including Mimosin—target docking were: (a) it shares a lesser number of protein targets with MARK4 target proteins and those share proteins are FYN, GFPT1, SLC6A1, and WEE1 and (b) it was not executed in SwissDock.

In the docking study, we observe that in the case of Apigenin, its Δ*G* is promising and in most of the targets, it is less than or equal to -7.29 kcal/mol, and the best binding is with CDK5 (where Δ*G* is -8.07kcal/mol). Fisetin shows the best binding possibility with PTK2 (Δ*G* = -8.08 kcal/mol), AURKB (Δ*G* = -8.16 kcal/mol), PTPRS (Δ*G* = -8.33 kcal/mol), CDK5 (Δ*G* = -8.45 kcal/mol), and comparatively lesser binding possibility with MAPT (Δ*G* = -6.52 kcal/mol). Gingerol has the highest binding possibility with MAPK3 (ΔG = -9.10), followed by CDK1, CDK2, CDK5, PDPK1, TYK2, JAK1, JAK2, and LIMK1, which have ΔG < -8 kcal/mol and >-9.0 kcal/mol). Quercetin shows the best binding possibility with CDK5, CSNK2A1, and PTPRS and displays comparatively lesser binding possibility with MAPT. In the case of Resveratrol, except MAPT, most of the proteins show close binding affinity. In the case of vanillin, the Δ*G* is within a closely related range (-6.14 kcal/mol -6.59 kcal/mol). Finally, we conclude that among all these drugs, Gingerol and Fisetin appear to be the highly promising drugs against MARK4-based targets, followed by Quercetin, Resveratrol, and Apigenin (**Table 2**).

### MARK4 Dominantly Affects the Genes Associated With HCC and the Critical Cellular Functions

**Figure 5:**
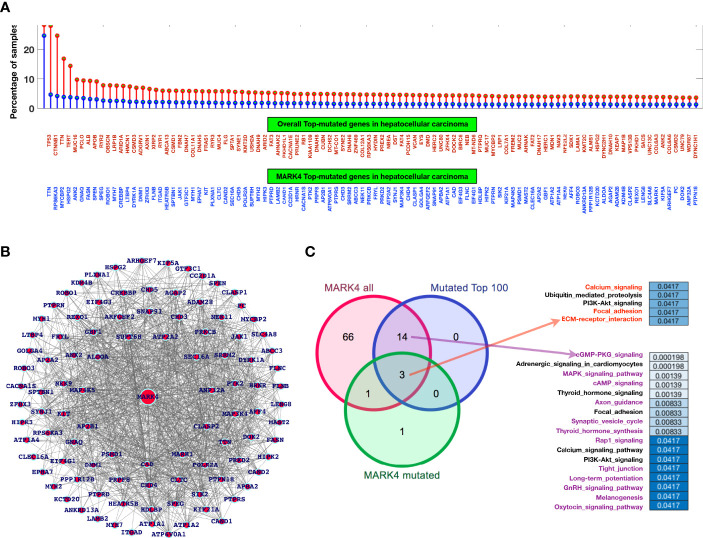
Mutational profiling and pathway-level understanding. **(A)** Top-ranked genes based on a mutation in the selected dataset for overall genes in the case of HCC and the genes common to MARK4-interactors. **(B)** Network representing top-ranked mutated genes associated with MARK4. **(C)** Venn diagram to map out the common and exclusively enriched pathways.

**Figure 6:**
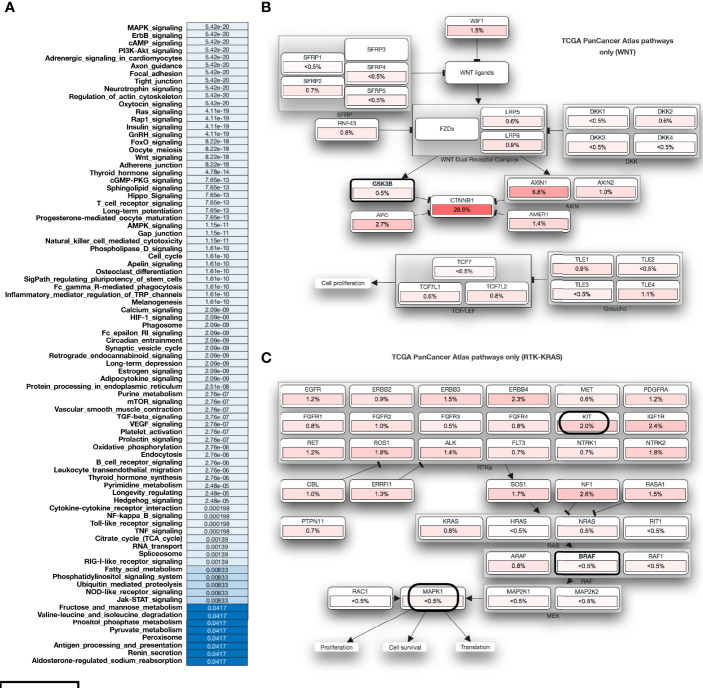
Functional impact of mutation in case of HCC. **(A)** Overall enriched pathways with their respective p-values in the case of HCC. **(B, C)** represents those mutated genes and belongs to the TCGA PanCancer Atlas pathways acting as major role players.

We have performed the mutational profiling of HCC genes, and to establish the linkage MAPK4 with human HCC. In addition, we mapped out those genes which are known to be the potential MARK4 targets and have presented top 100 genes in **Figure 5A**. The network of MARK4-associated HCC mutated genes are presented in **Figure 5B**. We performed pathway enrichment analysis of all the genes lists of all the MARK4, top 100 MARK4-associated mutated, and top 100 overall mutated genes and compared them by using Venn diagram plot (**Figure 5C**). Overall, calcium signaling, ubiquitin-mediated proteolysis, PI3K-Akt signaling, focal adhesion, and ECM-receptor interaction pathways were commonly enriched. There were 14 enriched pathways shared for the genes sets of MARK4 overall and mutated top 100 (**Figure 4C**). We have also presented the overall enriched pathways with their respective *p-values* for the HCC mutated genes as shown in **Figure 6A** and highlighted those genes that appear dominant in TCGA PanCancer atlas pathways (**Figures 6B, C**).

**Figure 7:**
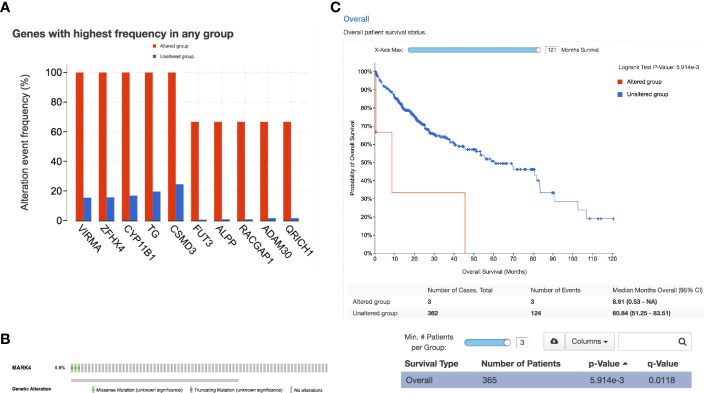
Clinical relevance. **(A)** Overall highly mutated genes in the case of HCC. **(B)** Percentage of patients showing a mutation in MARK4 and **(C)** survival curve representing the significance of MARK4 in the HCC clinical samples.

Finally, we have identified the genes with the highest mutation frequency (**Figure 7A**) and performed the overall impact of mutations in the MARK4 gene in the clinical samples available in the TCGA database (**Figure 7B**) and also performed the survival analysis. We observe that only 0.8% of patients show mutations in the MARK4 gene (**Figure 5E**), while in terms of its clinical relevance, it appears highly significant where the *p-value* was 5.914e-3 (**Figure 7C**). MARK4 co-expressed genes were also analyzed by using cBioPortal and the top-ranked co-expressed genes (mRNA expression of 367 samples correlated with MARK4) were presented in **Table 3**. These top-ranked co-expressed genes were GSK3A, BLOC1S3, BICRA, SCAF1, SIX5, STRN4, ZBTB45, CIC, ZNF628, XRCC1, CNOT3, ZNF335, ZNF574, PAK4, ARID3A, CLASRP, DMWD, DHX34, NECTIN2, FIZ1, CCDC97, PHYHD1, FBXO46, REXO1, and DMAC2.

**Table 3:** Top-ranked co-expressed genes for MARK4.

Correlated Gene	Cytoband	Spearman's Correlation	p-Value
**GSK3A**	19q13.2	0.534	6.03E-28
**BLOC1S3**	19q13.32	0.533	9.04E-28
**BICRA**	19q13.33	0.49	4.04E-23
**SCAF1**	19q13.3-q13.4	0.484	1.64E-22
**SIX5**	19q13.32	0.48	3.68E-22
**STRN4**	19q13.32	0.474	1.47E-21
**ZBTB45**	19q13.43	0.465	1.07E-20
**CIC**	19q13.2	0.459	3.6E-20
**ZNF628**	19q13.42	0.457	5.72E-20
**XRCC1**	19q13.31	0.457	6.22E-20
**CNOT3**	19q13.42	0.455	8.75E-20
**ZNF335**	20q13.12	0.453	1.38E-19
**ZNF574**	19q13.2	0.453	1.42E-19
**PAK4**	19q13.2	0.452	1.47E-19
**ARID3A**	19p13.3	0.451	1.75E-19
**CLASRP**	19q13.32	0.445	6.4E-19
**DMWD**	19q13.32	0.444	8.85E-19
**DHX34**	19q13.32	0.441	1.39E-18
**NECTIN2**	19q13.32	0.441	1.44E-18
**FIZ1**	19q13.42	0.44	1.85E-18
**CCDC97**	19q13.2	0.435	4.4E-18
**PHYHD1**	9q34.11	-0.43	1.29E-17
**FBXO46**	19q13.32	0.428	1.71E-17
**REXO1**	19p13.3	0.427	2.23E-17
**DMAC2**	19q13.2	0.426	2.46E-17

## Discussion

Cancer is a highly complex and global leading disease that severely impacts the worldwide human population and hepatocellular carcinoma is one of the leading cancer types (2, 4, 71, 72). Many plants produce naturally occurring secondary metabolites known for their anti-cancer activities and appear to be the leading source of new clinical drugs. MARK4 is thought to play various roles, including guiding neuronal migration, cell polarity, microtubule dynamics, apoptosis, and cell cycle regulation, specifically in the G1/S checkpoint, cell signaling, and differentiation, among many other complex cellular functions, according to previous detailed investigations (29–31, 36, 73, 74). MARK4 has recently been implicated in breast cancer cell proliferation and migration *via* blocking Hippo signaling. MARK4 is an important component of energy metabolism and homeostasis. Any changes in MARK4 expression can disrupt key cellular pathways, including mTOR and NF-kB, resulting in a variety of health problems. MARK4 is a key component of the Wnt signaling system that has been connected to prostate cancer caused by Wnt. By activating JNK1 and blocking the p38MAPK pathways, MARK4 promotes obesity and cell death. It also increases breast cancer cell proliferation and migration by inhibiting Hippo signaling (33, 36).

Overexpression of MARK4 is associated with various cancers, metastatic transitions, aberrant and uncontrolled neuronal migrations, and microtubule dynamics disturbance. MARK4 is connected to Hippo signaling, which plays a role in breast cancer growth and metastasis (32, 33, 35, 75). This kinase is abundant in glioblastomas and prostate cancer. MARK4 is thus a well-known therapeutic target in the fields of cancer, diabetes, and neurological disorders (25, 27, 76–78). It could also be a target for anti-cancer medication development. Glioma progression is slowed when MARK4 expression is inhibited. MARK4 inhibitors decrease the growth and proliferation of a range of cancer cell types, implying that they could help patients with cancer. These inhibitors decrease the growth and proliferation of various cancer cell types, emphasizing the significance of MARK4 inhibitors in improving the outcomes of MARK4-related cancers (32–36, 79). Based on previous studies, MARK4 has been identified as a potential therapeutic target for cancer and other disorders. MARK4 has been shown to increase microtubule dynamics and confer paclitaxel resistance in HCC, making it a good candidate for paclitaxel resistance therapy. Because it can directly connect with microtubules, MARK4 is a viable target for sensitizing HCC to paclitaxel treatment (80–82). In the previous studies, there are many relevant works. Still, they are in different directions. To simplify and bring many directional works in one study, we have applied an integrated approach from data acquisition to analysis and network-level understanding (63, 69, 83, 84).

From this study, there are many concluding points in terms of the MARK4 interactors, drug-target identifications, mutational profiling, functional aberrations, and clinical relevance, which are quite straightforward from data analysis to presentation, potential drug-target prediction, and conclusion. For drug-target prediction, we have selected Apigenin, Fisetin, Gingerol, Mimosin, Quercetin, Resveratrol, and Vanillin and predicted binding possibility by using SwissDock (18, 28, 50, 66, 85). Thus, we could say that in clinical samples, MARK4 appears highly relevant in terms of playing a role in the survival of HCC patients (as shown in **Figures 5E, F**), where the p-value looks very significant, which is 5.914e-3. From the co-expression data, we observe that the top-ranked co-expressed genes were GSK3A, BLOC1S3, BICRA, SCAF1, SIX5, STRN4, ZBTB45, CIC, ZNF628, XRCC1, CNOT3, ZNF335, ZNF574, PAK4, ARID3A, CLASRP, DMWD, DHX34, NECTIN2, FIZ1, CCDC97, PHYHD1, FBXO46, REXO1, and DMAC2.

In summary, the steps include prediction of MARK4 interactors, physicochemical properties evaluation of the herbal drugs, large-scale clinical sample analysis followed by the prediction of mutated genes, cross-linkage of MARK4 with clinical dataset, and finally the overall impact at functional level. To bridge the information gap between plant biologists and clinical researchers, this effort gave information on clinically successful plant-based anti-cancer treatments and underestimated yet potentially effective therapies. Furthermore, researchers have found it easier to produce a phytochemical as an effective anti-cancer medicine thanks to significant advances in synthetic chemistry, omics studies to pinpoint the target genes/proteins, and efficient drug delivery systems.

## Conclusions

Among the extremely top-ranked MARK4-interactors were YWHAE, CSNK2B, PPP2R1A, PPP1CA, TUBA1A, TUBB, MTOR, VDAC2, MYH10, ARFGEF2, RPTOR, NEDD4, PSMC2, SMARCA4, TUBG1, MYH9, PPP2CB, CDC42, NEDD4L, EIF2AK4, USP7, CDK8, PLK3, PRKCI, SCRIB, STK11, ARHGEF2, USP9X, and NUAK1 were among them and YWHAE, CSNK2B, PPP2R1A, PPP1CA, TUBA1A, TUBB, MTOR, VDAC2, MYH10, ARFGEF2, RPTOR, and NEDD4 are those proteins which connect the highest number of proteins listed here. Most of these proteins are associated with critical signaling pathways and biological functions. These critical pathways are MAPK signaling, PI3K-AKT signaling, FoxO signaling, Wnt signaling, TCR signaling, BCR signaling, NK cell-mediated cytotoxicity, TGF-beta signaling, and cytokines signaling pathways. The majority of these pathways are known to control different types of cancers and potentially HCC directly. Moreover, MARK4 shares the highest number of the target protein with Gingerol, followed by Resveratrol, Quercetin, Fisetin, Apigenin, and Vanillin. In contrast, Mimosin shares the least number of the target proteins (86–89).

## Data Availability Statement

The datasets presented in this study can be found in online repositories. The names of the repository/repositories and accession number(s) can be found in the article/supplementary material.

## Author Contributions

Conceptualization, SA, MM, MAd, MH, and MAb. Methodology, SA, MM, NA, MAd, and MAb. Software, MM. Validation, SA, MM, and MAb. Formal analysis, SA, NA, MM, and MAb. Investigation, SA, MM, and MAd. Resources, MM, and MAb. Data curation, SA, LA-K, MM, and MAb. Writing—original draft preparation, SA, MM, and MAb. Writing—review and editing, SA, MM, and MH. Visualization, SA and MM. Supervision, MM and MAb. Project administration, MAb. Funding acquisition, LA-K and MAb. All authors contributed to the article and approved the submitted version.

## Conflict of Interest

The authors declare that the research was conducted in the absence of any commercial or financial relationships that could be construed as a potential conflict of interest.

## Publisher’s Note

All claims expressed in this article are solely those of the authors and do not necessarily represent those of their affiliated organizations, or those of the publisher, the editors and the reviewers. Any product that may be evaluated in this article, or claim that may be made by its manufacturer, is not guaranteed or endorsed by the publisher.
